# Pelvic incidence measurement using a computed tomography data-based three-dimensional pelvic model

**DOI:** 10.1186/s13018-018-1050-4

**Published:** 2019-01-10

**Authors:** Hong-Fang Chen, Jie Mi, Heng-Hui Zhang, Chang-Qing Zhao

**Affiliations:** Shanghai Key Laboratory of Orthopaedic Implants, Department of Orthopaedics, Shanghai Ninth People’s Hospital, Shanghai Jiao Tong University School of Medicine, 639 Zhizaoju Rd, Shanghai, 200011 People’s Republic of China

**Keywords:** Pelvic incidence, Pelvic reconstruction, Sagittal balance, Spino-pelvic alignment, Sagittal parameter

## Abstract

**Objectives:**

To introduce a new method of pelvic incidence (PI) measurement based on three-dimensional (3D) pelvic models reconstructed from CT images and to report the normal distribution of PI in normal pelvic anatomy.

**Methods:**

CT images of 320 subjects with normal pelvic anatomy who visited the Radiology Department between 2006 and 2017 were retrospectively selected and saved in Digital Imaging and Communications in Medicine (DICOM) format. A computerized method was employed to determine the bony landmarks required for the measurement of PI. To quantify the method’s accuracy and reliability, the intraclass correlation coefficient (ICC) was calculated. A subgroup of 30 DICOM files was randomly selected to perform a validation study. Three independent testers performed all procedures. All measurements were performed twice independently by the three testers on all 10 subjects with an interval of 2 weeks. Independent samples *t* tests were used to identify statistically significant differences in the PI value between sexes. Pearson correlation coefficient was employed to determine the relationship between PI and age.

**Results:**

PI measurement using the new method resulted in an excellent intraobserver reliability (0.9612, range 0.8917–0.9893; *p* < 0.001) and interobserver reliability (0.9867, range 0.9611–0.9964; *p* < 0.001). PI was significantly different between sexes, with larger PI in women (*p* = 0.019). PI was significantly larger in the 40–80-year age group (45.94 ± 9.08°) than the < 40-year age group (43.50 ± 7.39°). We did not find any linear correlation between PI and age in the male (*r* = 0.140, *p* = 0.105) or female subgroup (*r* = 0.119, *p* = 0.107). A weak correlation between PI and age overall was observed (*r* = 0.142, *p* = 0.011).

**Conclusion:**

Accurate PI measurement could be achieved by a CT data-based 3D pelvic model.

## Introduction

Pelvic incidence (PI), a spinal sagittal parameter, is vital in assessing spinal balance and in the guiding operative principle [1]. PI is defined as an angle subtended by the line connecting the midpoint of the superior sacral endplate and hip axis (the midpoint of the line connecting the center of the left and right sides of femoral heads) and the line perpendicular to the midpoint of the sacral endplate [2]. As an anatomical parameter, PI is a constant value when one reaches bone maturity and is not affected by the spatial orientation of the pelvis, which is specific to every individual. Numerous studies proved that a close relationship between PI and several sagittal spinal parameters exists [3–6]. Moreover, PI has been used in predicting postoperative lumbar lordosis [7–9]. Postoperative PI-lumbar lordosis mismatch could result in a failed spinal surgery [10]. Alignment of the spino-pelvic complex needs to be carefully analyzed before spinal surgery to avoid adjacent segment disease, proximal junction kyphosis, and distal junctional kyphosis. Hence, accurate PI measurement is crucial before spinal surgery.

PI is typically measured using plain radiological images, which are obtained with patients in a standing position, with superimposition of both femoral heads and the anterior superior iliac spines (ASISs). However, with the projective nature of X-ray, anatomical structures could not be well observed in the standardized standing lateral X-ray of the spine and pelvis; thus, superimposition of both femoral heads and a perfect mid-sagittal view of the pelvis could not be achieved, thereby resulting in a large intra- and interobserver variation. The intra- and interobserver agreement rates with PI measurements via plain film X-ray were 0.84 and 0.79, respectively [11]. With the advancement of radiological imaging techniques, more accurate PI measurement is possible through three-dimensional (3D) images. Vrtovec et al. [12] proposed a method for measuring PI in 3D images obtained by CT in a normal population. The mid-sagittal plane and femoral heads could be clearly identified. However, the determination of the midpoint of the sacral endplate in their method could be oversimplified and inaccurate because of the irregularity of the sacral endplate. Therefore, our study aimed (1) to introduce a new method of PI measurement based on 3D pelvic models reconstructed from CT images, whose accuracy and reliability are quantified statistically, and (2) to report the normal distribution of PI in 320 Chinese subjects with normal pelvic anatomy, including the correlation between PI and age, and differences between the sexes.

## Patients and methods

### Subjects

In this retrospective study, we searched our image database for subjects who visited the Radiology Department between 2006 and 2017 and received a CT scan for pelvic injury or acute abdominal disease. Inclusion criteria were as follows: (1) age between 20 and 80 years old; (2) complete pelvic imaging, including the iliac crest and ischial tuberosity; (3) no fracture of the spine, pelvis, or femur; and (4) no history of spine, pelvic, or hip surgery. Exclusion criteria were as follows: (1) any anatomical anomalies of the pelvis, spine, or femur, including lumbar spondylolisthesis, developmental dysplasia of the hip, lumbar sacralization or lumbarization of the first sacral vertebra, and malunion of lumbosacral fractures; (2) a history of any conditions that may affect bone growth, including cerebral palsy, Graves’ disease, diabetes mellitus, and Cushing disease; and (3) severe osteoporosis or arthritis. The radiographic images of 3226 patients were reviewed, and 320 subjects were included in the final study cohort. CT scans were obtained using Siemens SOMATOM Definition Flash 128 scanners (Somatom Definition FLASH, Siemens Healthcare, Forchheim, Germany). The mean voxel size was 0.98 × 0.98 × 1.00 mm. This study was approved by our hospital’s institutional review board [2016141]. Informed consent was waived in this study. Imaging data were exported as Digital Imaging and Communications in Medicine (DICOM) files for further analysis.

### Pelvic incidence measurement

**Fig. 1:**
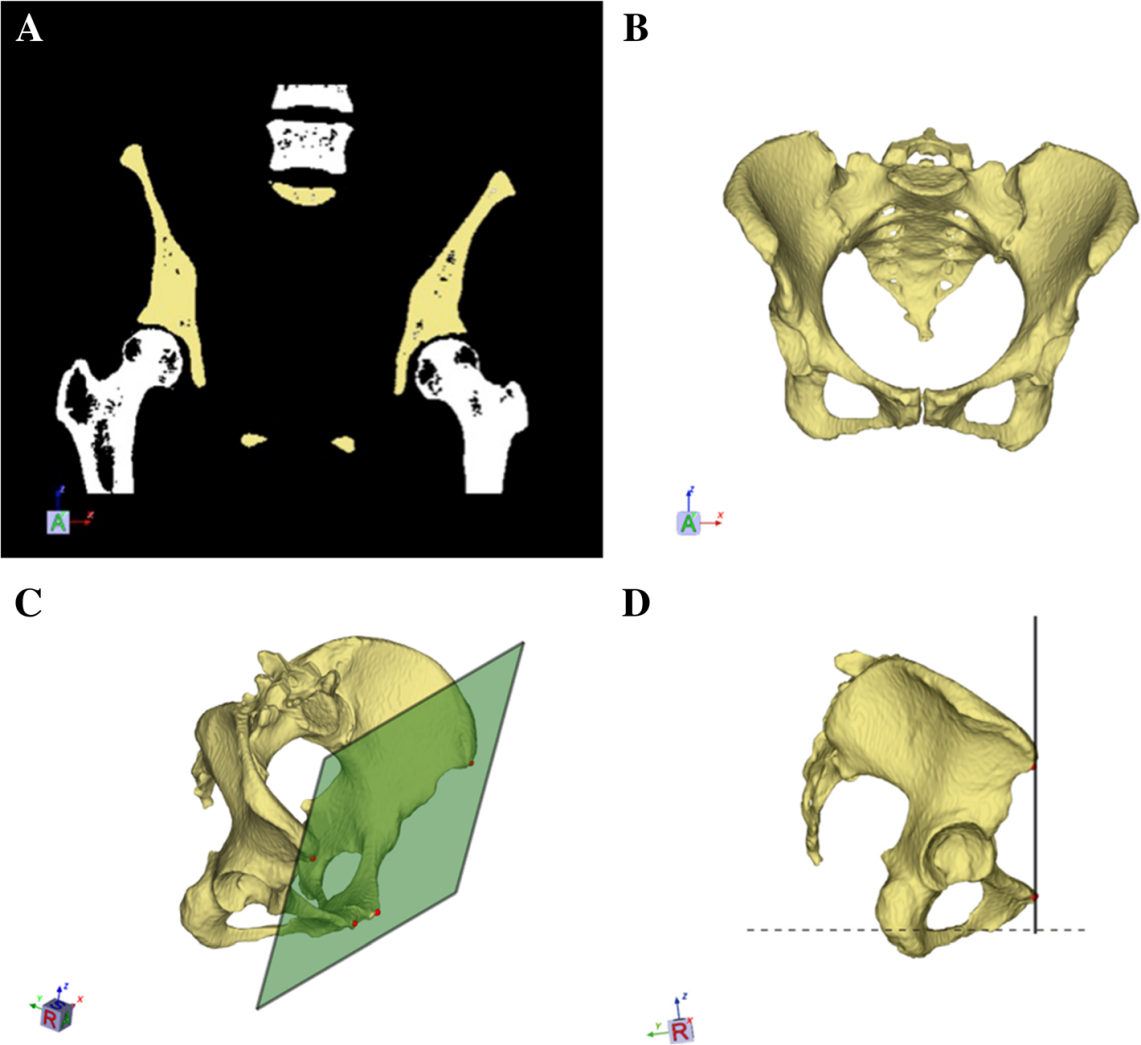
**a**–**d** Segmentation, modeling, and alignment of the 3D pelvic model. **a** A spherical mask was fitted to isolate the pelvis from CT volume images. Femurs and the lumbar vertebra were manually removed. **b** A virtual 3D pelvic model was automatically reconstructed using a threshold and region-growing algorithm. **c** Both anterior superior iliac spines and pubic tubercles were manually selected to determine the anterior pelvic plane. **d** The pelvic model with the anterior pelvic plane perpendicular to the horizontal plane

CT images of the subjects were saved in DICOM format. The DICOM files were imported into the SPINEPARA software, which was developed in house by our engineers. After threshold and region-growing segmentation of the bone structure images, both femoral heads and vertebrae were manually removed. The threshold in this study is based on Hounsfield unit (HU) method, which is widely used to asses bone mineral density. The threshold was decided by two independent spine surgeons, and an appropriate HU value that best delineate the shape of the pelvis as well as the sacral endplate was agreed on as the specific threshold for each patient. Subsequently, the isolated 3D pelvic surface mesh models were reconstructed; a high-performance scalable isocontouring algorithm was applied. Each pelvic model was minimally smoothed without surface simplifications to maintain the natural surface profile. The pelvis was positioned according to the anterior pelvic plane (APP) proposed by Lewinnek et al. [13]. To determine the APP, four bony landmarks in the pelvic model were manually selected: both pubic tubercles (PTs) and ASISs. The midpoint of the PTs and the most ventral aspect of the ASISs were automatically determined using a unique iterative algorithm. After APP determination, we realigned the model with superimposition of the ASISs and acetabular cups to obtain a perfect sagittal view, with the APP perpendicular to the horizontal plane (Fig. 1a–d).

**Fig. 2:**
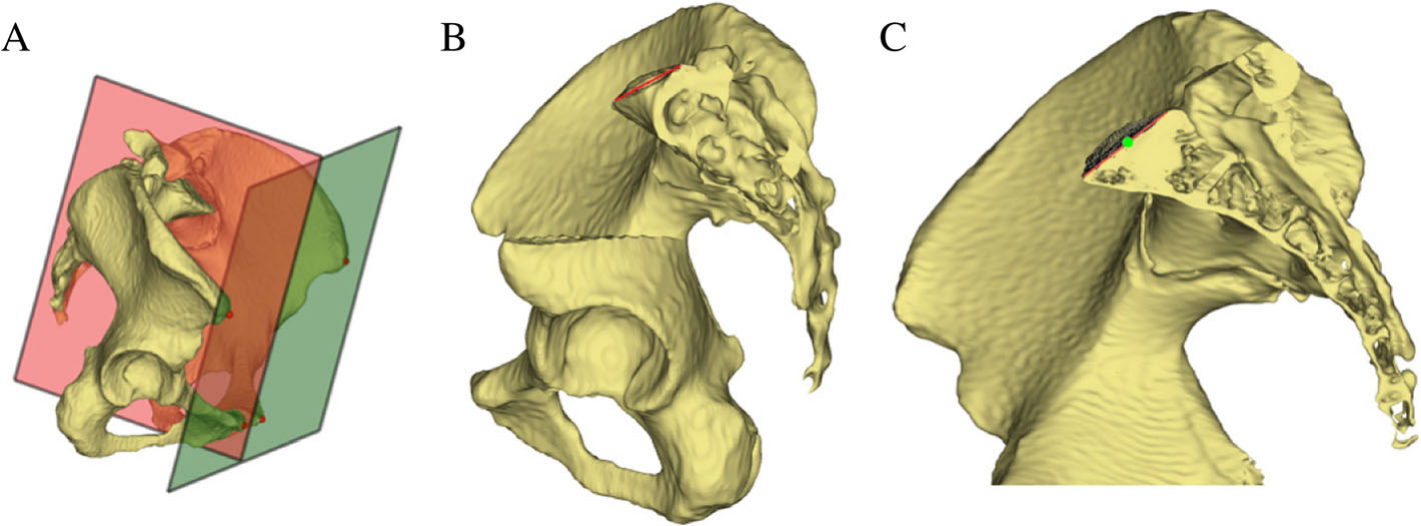
**a**–**c** The schematic shows the determination of the best-fit sagittal line segment and its midpoint of the sacral endplate. **a** The calculation of the mid-sagittal plane used an Iterative Closest Points algorithm based on the anterior pelvic plane. **b** Approximately 5000–8000 surface points (black) on the sacral endplate were automatically extracted to fit a sagittal line segment. For a better view of the sacrum, part of the left iliac bone was removed. **c** The midpoint (green) of the sagittal line segment was calculated by the software. For a better view of the sagittal line segment, the left part of the pelvis was removed

**Fig. 3:**
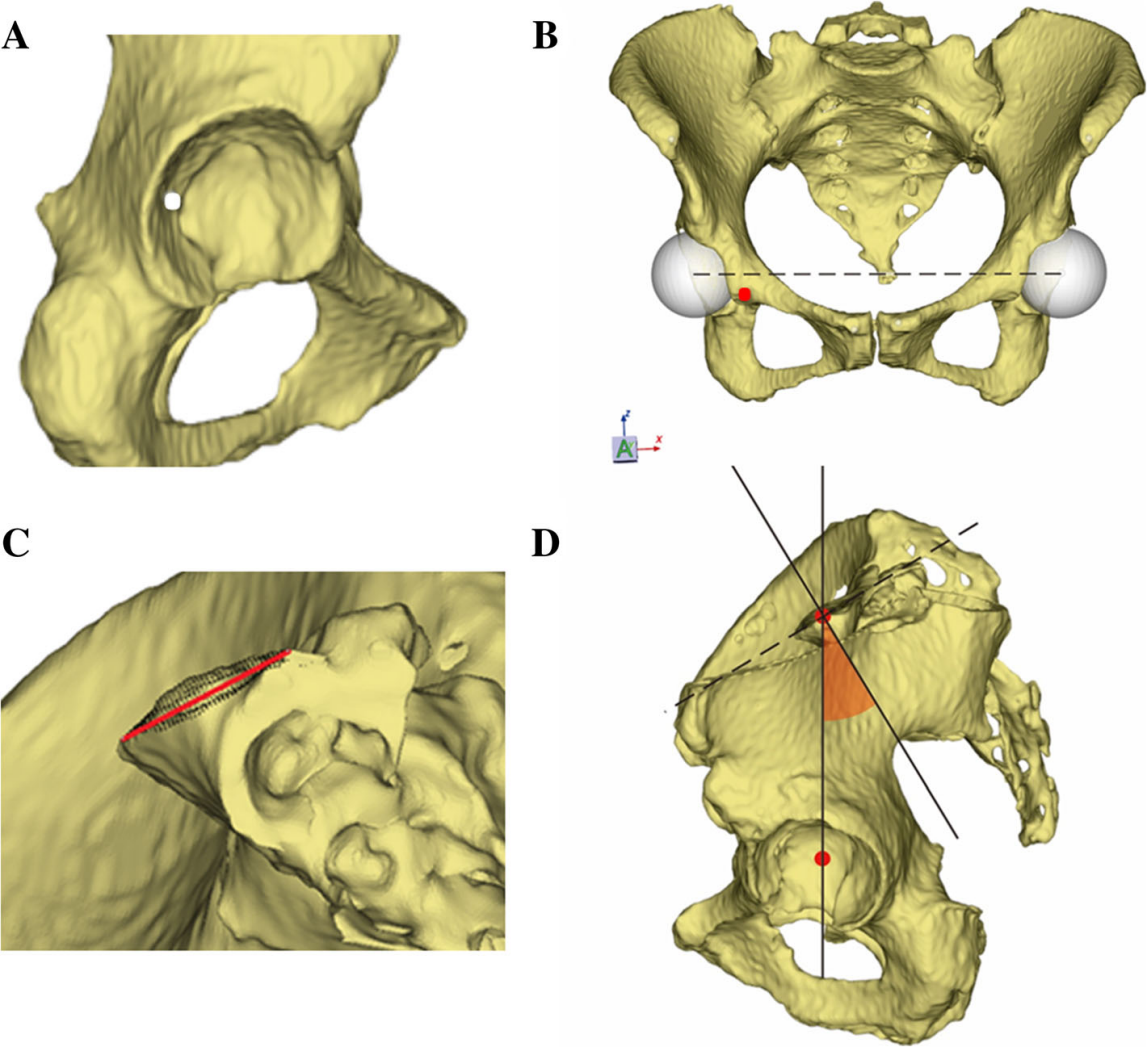
**a**–**d** The schematic shows the measurement of pelvic incidence. **a** one point (white) was manually located at the left acetabular fossa to initiate the analysis. **b** Two spheres that best fit to the acetabular fossae were generated. The center of both spheres and hip axis were calculated by the software. **c** Surface points (black) were located on the sacral endplate, and a sagittal line segment that best fits to the sacral endplate was extracted from the surface points. **d** The midpoint of the sacral endplate was automatically determined by the software and pelvic incidence was calculated as the angle between the line perpendicular to the midpoint of the sacral endplate and the line connecting the midpoint with the hip axis

**Fig. 4:**
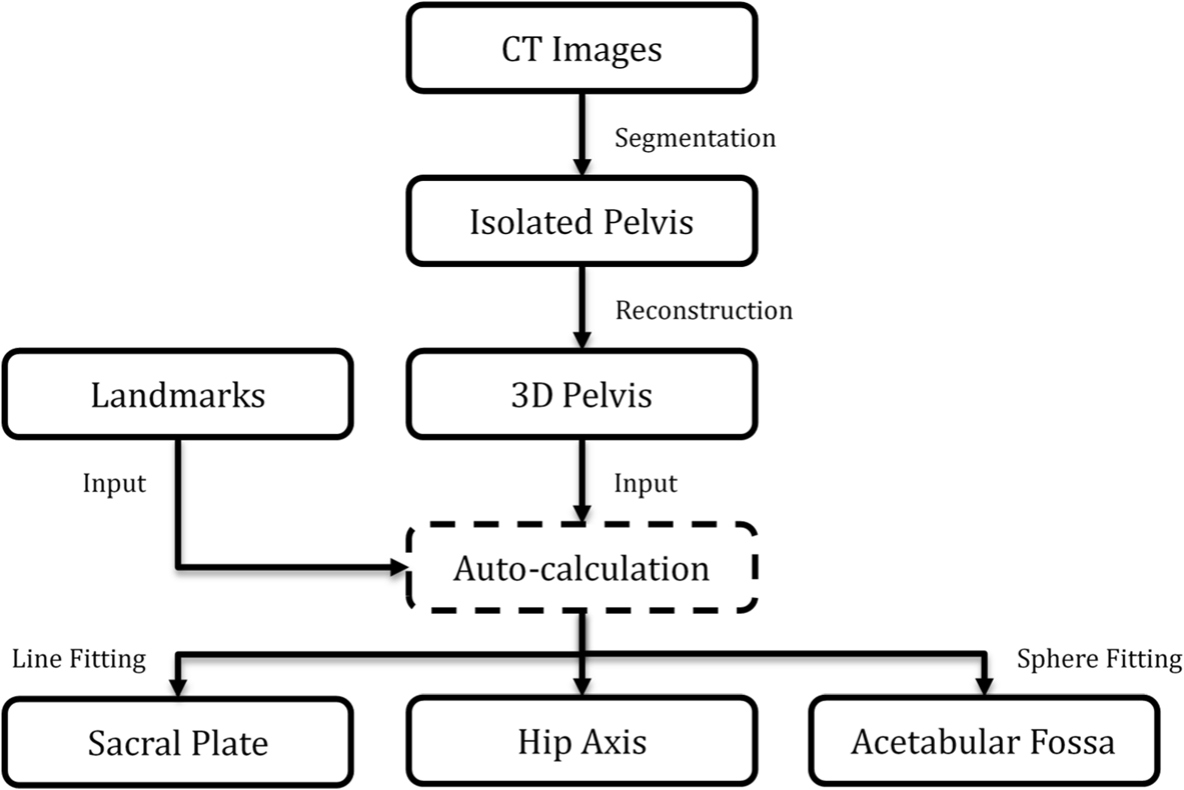
The schematic shows the entire process of measuring pelvic incidence via a computed tomography data-based three-dimensional pelvic model

To calculate PI in 3D models, at least two anatomical references have to be determined, that is, the hip axis and the midpoint of the sacral endplate. Two points were manually selected in the pelvic models at an approximate location of the right and left acetabular cups to initialize the pelvis measurement module in software. The software automatically determined two spheres that best fit to the acetabular fossa and automatically calculated the exact centers of the spheres as the centers of the femoral heads. The midpoint of the line connecting the center of the two spheres was the hip axis. Moreover, approximately 5000–8000 surface points around the initial midpoint located on the surface of the sacral endplate were automatically extracted and projected onto the mid-sagittal plane, which was calculated using the Iterative Closest Points algorithm. And the number of the surface points was decided by the variation of area of endplates. These in-plane sagittal points were processed to fit a sagittal line segment using the least squares method (Fig. 2a–c). The midpoint of the sacral endplate with anterior and posterior end points was determined. Lastly, PI was calculated as the angle between the line perpendicular to the midpoint of the sacral endplate and the line connecting the midpoint with the hip axis (Fig. 3a–d). Figure 4 shows the entire process of our method.

### Measurement validation

A subgroup of 10 DICOM files was randomly selected to validate the measurement in this study. Three independent testers performed all procedures, including CT image navigation, pelvic model reconstruction, and bony landmark selection. All measurements were performed twice independently by the three testers on all 10 subjects with an interval of 2 weeks. To compare this new method with the traditional method, a subgroup of 10 full-length spine radiographs was randomly selected in our image database. Intra-and interobserver reliability were calculated as aforementioned.

### Statistics analysis

The intraobserver and interobserver reliabilities of the measurements were determined using the intraclass correlation coefficient (ICC). To accommodate an estimated ICC of 0.9 and a desired 95% confidence interval (95% CI; *α* = 0.05, power 80%) with a width of 0.2°, two-way ANOVA was used to calculate the interobserver ICC, and two-way mixed-effects ANOVA was applied to calculate the intraobserver ICC. Kolmogorov-Smirnov test was employed to determine whether the PI values were normally distributed in the male and female cohorts. Differences in PI values between male and female cohorts were evaluated by a pooled-variance or separate-variance *t* test. Levene test was used to determine whether pooled-variance or separate-variance *t* test should be performed. Moreover, Pearson correlation coefficient was used to determine the relationship between PI and age in the normal Chinese subjects. Statistical analysis was performed using the Statistical Package for the Social Sciences (SPSS) for windows, version 22 (Armonk, NY: IBM Corp). Differences were considered significant when *p* was < 0.05.

## Results

### Measurement validation

PI measurement with our method had an excellent intraobserver reliability (0.9612, range 0.8917–0.9893; *p* < 0.001) and interobserver reliability (0.9867, range 0.9611–0.9964; *p* < 0.001). PI measurement via full-length spine radiographs had a fair intraobserver reliability (0.864, range 0.661–0.961; *p* < 0.001) and interobserver reliability (0.897, range 0.734–0.971; *p* < 0.001).

### Subjects

The final study cohort included 320 subjects (mean age 46.17 years, standard deviation [SD] 15.84 years, range 20–80 years), of which 135 were men (mean age 43.81 years, SD 16.03 years, range 20–77 years) and 185 were women (mean age 47.88 years, SD 15.52 years, range 20–80 years).

### Pelvic incidence measurement

PI measured by our dedicated computerized software is presented as a mean ± SD. The resulting PI values were 43.67 ± 8.00° (range 30.49–64.08°) for male subjects, 45.92 ± 8.78° (range 30.78–70.12°) for female subjects, and 44.97 ± 8.52° (range 30.49–70.12°) for both sexes. As the PI values in both male and female subjects followed a normal distribution (*p* > 0.05) and the Levene test showed that the variances in the PI value between two sexes were not significantly different (*p* = 0.272), a pooled-variance *t* test was adequate in comparing the two groups. A significant difference in the PI value between male and female subjects was found (*p* = 0.019), and no linear correlation between PI and age in both sexes was observed in this study. PI was significantly larger in the 40–80-year age group (45.94 ± 9.08°) than 20–40-year age group (43.50 ± 7.39°) (Table 1).

**Table 1:** Correlation between pelvic incidence and age

	All group		< 40 years old		40–60 years old		> 60 years old	
	*N* = 320		*N* = 127		*N* = 128		*N* = 65	
	Mean	SD	Mean	SD	Mean	SD	Mean	SD
Age	46.17	15.84	29.14	5.58	52.26	5.19	67.43	5.30
PI	44.97	8.52	43.50	7.39	45.98	8.78	45.86	9.70
r	0.142*		0.094		− 0.055		0.220	
*p* value	0.011		0.293		0.539		0.078	

*Statistically significant correlation coefficient (*p* < 0.05)

## Discussion

It is generally accepted that PI remains constant during adulthood regardless the motion of sacroiliac joint and is not affected by the spatial orientation of the pelvis. However, this point of view is likely to be challenged. Some studies reported that PI continues to increase linearly after skeletal maturity and throughout an individual’s lifespan [14–16]. Vrtovec et al. [17] reported that PI continued to increase linearly after skeletal maturity, which may be due to the weight-bearing wear of the acetabular cartilage and subsequent remodeling process affecting the pelvis that leads to an anterior drift of the acetabulum relative to the sacrum in the aging process. Legaye [18] attributed the correlation between age and PI in adults to the destabilized sacroiliac joints, with anatomical forward rotation of the sacrum, which led to increased PI. Howard [19] recruited 50 healthy volunteers to investigate the change of PI between three pelvic positions: maximal anterior pelvic rotation, maximal posterior pelvic rotation, and resting baseline pelvic posture. And the results suggested that 80% of the subjects changed PI by a mean of 2.76°, 26% changed > 5°. It may indicate that measurable motion at the sacroiliac joints cannot be neglected even in healthy population. In this study, we only found a weak correlation (*r* = 0.142, *p* < 0.05) between age and PI in the whole group. In our study, we did find that significant difference in PI between sexes with larger PI in females, which is consistent with the findings of previous studies [20, 21]. We also found PI was significantly larger in the 40–80-year age group (45.94 ± 9.08°) than < 40-year age group (43.50 ± 7.39°). Bao et al. [22] demonstrated significant PI differences between genders with higher PI in female subjects, and PI was larger in older patients, especially those over 45 years old. They also revealed that gender, age, and malalignment were associated factors for increased PI. Whether increased PI is caused by anterior drift of the acetabulum due to coxal bone remodeling or sacral rotation due to sacroiliac destabilization, studies by now cannot determine which plays a major role, probably both process are involved. Longitudinal studies would shed new light on this topic.

Duval-Beaupère et al. [23] introduced the classic method for measuring PI on plain radiographic images. In their method, the length of the sacral endplate and the edge of the femoral head had to be manually drawn in two-dimensional (2D) radiographs. The midpoint of the line connecting the center of the femoral heads is considered the hip axis. However, with the projective nature of 2D radiograph, superimposition of the femoral heads and a perfect mid-sagittal view of the pelvis are difficult to obtain. Although software has been developed, such as SURGIMAP (Nemaris Inc., New York, NY), to calculate the value of PI automatically after anatomical points are manually selected, PI measurement may be inaccurate in 2D sagittal radiographs and considered to have a large intra-/interobserver variation [11].

Vrtovec et al. [12] reported a method for measuring PI in CT images using multiplanar reformation. Their process of determining the hip axis was similar to that of ours. Briefly, they selected two points at the location of the right and left femoral heads, and the computerized method determined the spheres that best fit the edges of the two femoral heads. However, their calculation of the exact center of the sacral endplate was totally different. They defined the center of the sacral endplate as the midpoint of the line passing through the anterior and posterior edges as well as the left and right edges of the end plate. The inclination of the sacral endplate in their method was determined from the plane that best fits the endplate. However, the morphology of the superior sacral endplate is not considered a regular circle anatomically. In some reports, the sacral endplate is not even flat [24–26].

**Fig. 5:**
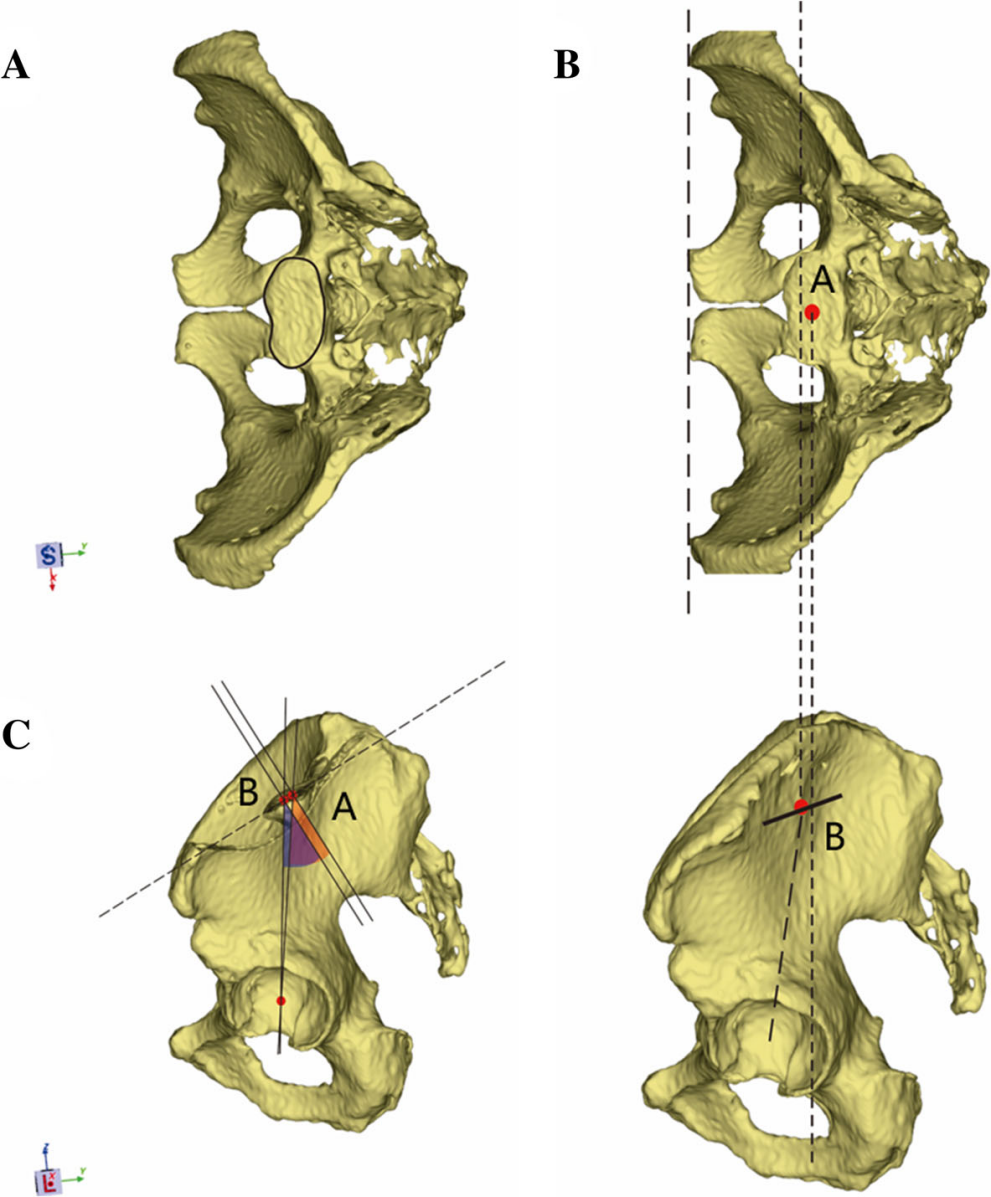
**a**–**c** The schematic shows the type 1 sacral endplate and the measurement of pelvic incidence based on CT and plain film X-ray methods. **a** Type 1 sacral endplate with a concave side anteriorly. **b** Point A is obtained as the midpoint of the segment crossing the mid-sagittal plane of the endplate via CT. Point B is obtained as the midpoint of the projection line via plain film X-ray method. **c** Point A is behind point B. Thus, the pelvic incidence value measured on CT is larger than that measured via plain film X-ray

**Fig. 6:**
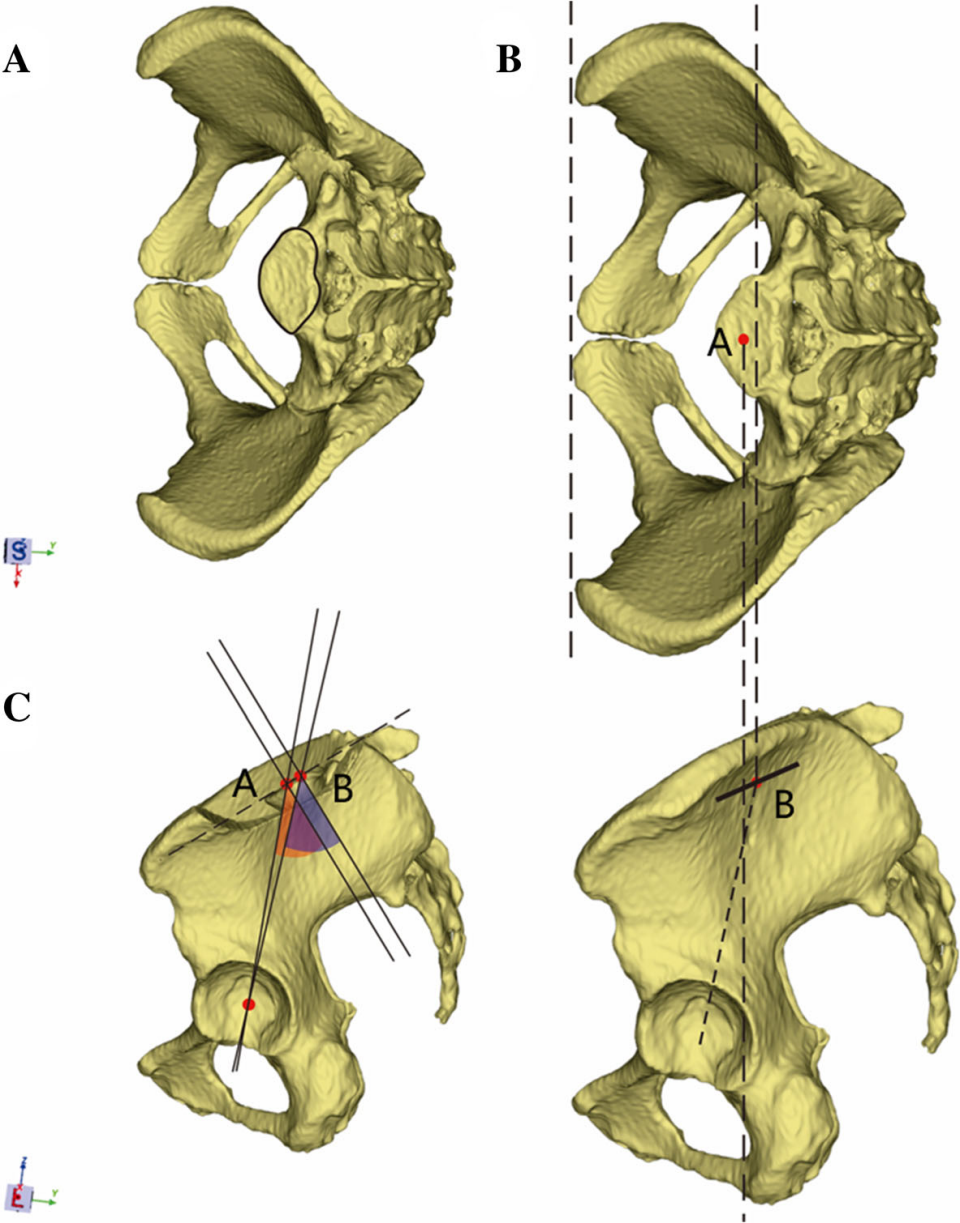
**a**–**c** The schematic shows the type 2 sacral endplate and the measurement of pelvic incidence based on CT and plain film X-ray methods. **a** Type 2 sacral endplate with a concave side posteriorly. **b** Point A is obtained as the midpoint of the segment crossing the mid-sagittal plane of the endplate via CT. Point B is obtained as the midpoint of the projection line via plain film X-ray method. **c** Point B is behind point A. Thus, the pelvic incidence value measured on CT is smaller than that measured via plain film X-ray

In this study, we describe two types of sacral endplates that are both normal variants of the sacrum. Nevertheless, with further simplification, we hypothesize that the superior sacral endplate is flat. We describe the sacral endplate with a concave side anteriorly as a type 1 sacral endplate; the midpoint of the segment crossing the MSP of the endplate is behind the midpoint of the projection line on plain film X-ray (Fig. 5a–c). Thus, the PI value obtained from the MSP is higher than that obtained via the projection theory. Moreover, we describe the sacral endplate with a concave side posteriorly as a type 2 sacral endplate; the midpoint of the segment crossing the MSP of the endplate is in front of the midpoint of the projection line on plain film X-ray (Fig. 6a–c). Consequently, the PI value obtained from the MSP is smaller than that obtained via the projection theory. In this study, we found 23 subjects can be classified as a type 1 sacrum, and 123 subjects can be classified as a type 2 sacrum.

Furthermore, Jean-Marc et al. [27] reported a PI of 52.4 ± 10.8° in 709 normal subjects, which was measured on standing whole-spine radiographs. Labelle et al. [24] reported a PI of 52 ± 5° in 160 normal subjects, which was measured by 2D X-ray. Vrtovec et al. [12] reported a normative PI value of 47 ± 10° in 370 normal subjects, which was obtained on CT images using the segment across the MSP of the sacral endplate and was underestimated at approximately around 5°. Thus, we postulate that type 2 sacral endplate is possibly more prevalent in the normal population. Nevertheless, this should be further validated in an anatomic study in the normal population.

In our method, we projected the superior sacral endplate on the MSP of the pelvis with perfect superimposition of the ASISs and acetabular cups. Determination of the superior sacral endplate in our measurement may reflect the 3D anatomical structure of the sacrum to a certain degree, and our measurement method could be applied to the sacrum with anatomical anomaly. However, our study does not imply that PI should not be measured using plain film X-ray and that we challenge the routine clinical protocol. In most studies, PI and other spinal sagittal parameters were obtained using plain radiographs, and the clinical usefulness of such approach has been validated [28, 29]. Our study also aimed to demonstrate that under certain circumstances, PI measurement in 2D cross-sections may not reflect the exact anatomical structures of the pelvis and that the PI value may be inaccurate.

Our study has some limitations. First, CT was performed with the patient in the supine position. Thus, only the anatomical parameters could be measured, whereas information on positional pelvic parameters, such as SS and PT, could not be obtained. Second, few patients received both total spine X-ray examination and pelvic CT scan; thus, we could not compare our method to the traditional plain film technique. These two methods will be validated in a further study. Third, some pelvic bony landmarks could not be accurately differentiated in the 3D reconstruction model. Fourth, approximately 8 to 10 min is required to perform a complete measurement of PI on a single subject. Moreover, it could be more time-consuming if the pelvis is difficult to isolate from CT images. Lastly, because of the retrospective nature and relatively small study cohort in our study, the mean PI value could not represent the whole Chinese population (most of our patients were from east China). Despite these limitations, our study is of great value, as we have provided a novel method of measuring PI on a 3D reconstruction pelvic model, especially in terms of the technique in determining the superior sacral endplate.

## Conclusion

Accurate PI measurement using a CT data-based 3D pelvic model is possible. We proposed a novel approach to determine the midpoint of the sacral endplate. In this study, we found a significant difference in PI between sexes, with larger PI in women, PI was significantly larger in the 40–80-year age group than the < 40-year age group, and no linear correlation between PI and age in the male or female subgroup. However, a weak correlation was observed between PI and age in the 320 Chinese adults.

## Abbreviations

3D: Three-dimensional
APP: Anterior pelvic plane
ASIS: Anterior superior iliac spine
CT: Computed tomography
DICOM: Digital Imaging and Communications in Medicine
ICC: Intraclass correlation coefficient
MSP: Mid-sagittal plane
PI: Pelvic incidence
PT: Pubic tubercle
SD: Standard deviation
SS: Sacral slope
